# Correlates of sedentary behavior in the general population: A cross-sectional study using nationally representative data from six low- and middle-income countries

**DOI:** 10.1371/journal.pone.0202222

**Published:** 2018-08-10

**Authors:** Ai Koyanagi, Brendon Stubbs, Davy Vancampfort

**Affiliations:** 1 Research and Development Unit, Parc Sanitari Sant Joan de Déu, Universitat de Barcelona, Fundació Sant Joan de Déu, Sant Boi de Llobregat, Barcelona, Spain; 2 Instituto de Salud Carlos III, Centro de Investigación Biomédica en Red de Salud Mental, CIBERSAM, Madrid, Spain; 3 Physiotherapy Department, South London and Maudsley NHS Foundation Trust, Denmark Hill, London, United Kingdom; 4 Health Service and Population Research Department, Institute of Psychiatry, Psychology and Neuroscience, King’s College London, De Crespigny Park, London, United Kingdom; 5 Faculty of Health, Social Care and Education, Anglia Ruskin University, Chelmsford, United Kingdom; 6 Leuven Department of Rehabilitation Sciences, Leuven, Belgium; 7 University Psychiatric Centre KU Leuven, Kortenberg, Belgium; University of Adelaide School of Medicine, AUSTRALIA

## Abstract

**Background:**

Sedentary behavior (SB) is associated with adverse health outcomes independent of levels of physical activity. However, data on its correlates are scarce from low- and middle-income countries (LMICs). Thus, we assessed the correlates of SB in six LMICs (China, Ghana, India, Mexico, Russia, South Africa) using nationally representative data.

**Methods:**

Cross-sectional, community-based data on 42,469 individuals aged ≥18 years from the World Health Organization’s Study on Global Ageing and Adult Health were analyzed. Self-reported time spent sedentary per day was the outcome. High SB was defined as ≥8 hours of SB per day. The correlates (sociodemographic and health-related) of high SB were estimated by multivariable logistic regression analyses.

**Results:**

The overall prevalence (95%CI) of high SB was 8.3% (7.1–9.7%). In the overall sample, the most important sociodemographic correlates of high SB were unemployment and urban residence. Physical inactivity, morbid obesity (BMI≥30.0 kg/m^2^), higher number of chronic conditions, poor self-reported health, higher disability levels, and worse health status in terms of mobility, pain/discomfort, affect, sleep/energy and cognition were associated with high SB. Several between-country differences were found.

**Conclusion:**

The current data provides important guidance for future interventions across LMICs to assist sedentary people to reduce their SB levels.

## Introduction

Sedentary behavior (SB) is defined as behaviors that involve sitting or reclining positions and low levels of energy expenditure (≤1.5 metabolic equivalents) during waking hours [1]. There is emerging evidence that SB is associated with adverse health outcomes across the lifespan, from adolescents [2] through to older adults [3]. Specifically, SB has been associated physical health conditions including obesity, type 2 diabetes, cardiovascular disease, and increased cardiovascular-specific and overall premature mortality [4–6]. Moreover, more recent evidence has suggested that SB is associated with some mental health comorbidities such as depression [7] and anxiety [8]. Some authors have suggested that the adverse relationship between SB and health outcomes may be independent of a person’s physical activity levels [9, 10].

Given the aforementioned deleterious outcomes, there has been an increasing emphasis on research aimed at reducing SB among adults in the last decade. A meta-analysis [11] showed that lifestyle interventions were able to reduce SB by 24 min/day (95%CI = 8 to 41 min/day, n = 3981) and in terms of interventions focusing on SB only, SB was reduced by 42 min/day (95%CI = 5 to 79 min/day, n = 62).

Whilst interventions focusing on SB may appear to show promise, more high quality research is needed to determine if such interventions are sufficient to produce clinically meaningful and sustainable reductions in sedentary time. An important step to aid the development of effective interventions, is a clear understanding and comprehensive examination of modifiable correlates of SB, which can be targets for effective interventions, while non-modifiable variables are important for targeting specific subgroups at increased risk. The focus to date on variables that might influence SB has mostly been on individual level correlates such as biological, psychological and behavioral factors [12]. However, it has become apparent that these are not stand-alone factors and addressing them in isolation will not result in a significant change in SB. Social, environmental and policy factors should also be taken into account. The current rationale is that factors that influence SB can be conceptualized using models such as the socio-ecological model [13, 14]. The socio-ecological model suggests that multiple relevant attributes influence health behavior. These include intrapersonal (demographic, biological, psychological, emotional and cognitive), interpersonal/ cultural (e.g., social support), physical environment (e.g., distance to services and facilities, financial costs, enjoyable scenery), and policy (laws, rules, regulations, codes) factors. Previous studies from the general population have provided some evidence that older age, female gender, lower education, full-time employment status, a higher body mass index (BMI), a higher income/socio-economic status, smoking, and the presence of depressive symptoms [13–15] are associated with more SB. Also, previously reported environmental factors associated with more SB include lack of proximity of green space, lack of neighborhood walkability, an unsafe environment, and bad weather conditions [13–15].

A major deficit in the literature is that the current evidence regarding SB is predominantly derived from studies conducted in high-income countries [15]. Given the markedly different occupational and socio-cultural structures, methods of transportation, and environmental factors (e.g., safety, climate) in in low- and middle-income countries (LMICs), there is a need for context specific research in these settings [16]. Another important point is that almost three-quarters of non-communicable disease-related deaths occur in LMICs. Thus, there is considerable potential for preventive interventions such as reducing SB in this neglected region in the world [17]. This is especially important given that compared to high-income countries, many people in LMICs, where two-thirds of the world’s population resides, have a much lower capacity to pay for adequate healthcare. While 90% of the global burden of disease is concentrated in LMICs, only 12% of global health spending takes place in LMICs [18]. Moreover, recently, several LMICs have started to adopt national policies or action plans to increase physical activity levels, but recommendations on how to reduce SB are currently lacking [19].

Another important point is that there is generally a paucity of large-scale multinational studies exploring SB correlates. Clearly, undertaking multinational studies enables exploration of SB correlates irrespective of national policies as well as available services and facilities, and at the same time allow comparison between countries in order to speculate the role of such factors in different countries.

Thus, given the aforementioned gaps and limitations within the literature, we aimed to investigate SB correlates among community-dwelling adults in six LMICs (China, Ghana, India, Mexico, Russia, South Africa) that participated in the Study on Global Ageing and Adult Health (SAGE). The selection of the correlates of SB (socio-demographics, health behavior, mental and physical health) was based on past literature [13–15]. A secondary aim was to compare differences in SB correlates across countries. The six countries included in our study comprise a large proportion of the world population, and are representative of diverse geographical locations and socioeconomic levels.

## Materials and methods

We conducted secondary data analysis of the Global Ageing and Adult Health (SAGE) survey which was conducted in China, Ghana, India, Mexico, Russia, and South Africa between 2007 and 2010. The World Bank classification at the time of the survey for the included countries were the following: Ghana (low-income country); China and India (lower middle-income countries although China became an upper middle-income country in 2010); Mexico, South Africa, Russia (upper middle-income countries). The dataset is publically available via the WHO website (http://www.who.int/healthinfo/sage/en/) where the questionnaire of this survey can also be found. Details of the survey methodology have been provided previously [20, 21]. In brief, in order to obtain nationally representative samples, a multi-stage clustered sampling design method was used. The sample consisted of adults aged ≥18 years while those aged ≥50 years were oversampled. Trained interviewers conducted face-to-face interviews. The survey response rates were: China 93%; Ghana 81%; India 68%; Mexico 53%; Russia 83%; South Africa 75%. Sampling weights were constructed to adjust for the population structure and non-response. Details on sampling weights can be found elsewhere [21]. Ethical approval for this survey was obtained from the WHO Ethical Review Committee and local ethics research review boards: Shanghai Municipal Centre for Disease Control and Prevention, Shanghai, China; Ghana Medical School, Accra, Ghana; International Institute of Population Sciences, Mumbai, India; National Institute of Public Health, Cuernavaca, Mexico; School of Preventive and Social Medicine, Russian Academy of Medical Sciences, Moscow, Russia; and Human Sciences Research Council, Pretoria, South Africa. All participants provided written informed consent.

### Sedentary behavior (Outcome variable)

Participants were asked to state the total time they usually spent (expressed in minutes per day) sitting or reclining including at work, at home, getting to and from places, or with friends (e.g., sitting at a desk, sitting with friends, travelling in car, bus, train, reading, playing cards or watching television). This did not include time spent sleeping. SB was assessed as a categorical variable [<8 or ≥8 hours per day (high SB)] [22].

### Socio-demographic variables

These included age (18–44, 45–64, ≥65 years), sex, highest level of education achieved (completed secondary or less), wealth, marital status (married/cohabiting or else), living arrangement (alone: Y/N), setting (urban or rural), and employment status (engaged in paid work ≥2 days in last 7 days: Y/N), Wealth quintiles were created based on country-specific income.

### Health behavior

These comprised of smoking (never, quit, current), alcohol consumption (never, non-heavy, heavy [23]), and physical activity. The validated Global Physical Activity Questionnaire was used to assess levels of physical activity [24]. Physical inactivity (i.e., not meeting the recommended guidelines) was defined as <150 min of moderate-to-vigorous physical activity in a typical week [25].

### Physical health

A stadiometer was used to measure height, while weight was measured with a routinely calibrated electronic weighting scale. Body mass index (BMI) was calculated as weight in kilograms divided by height in meters squared, and was categorized as <18.5 (underweight), 18.5–24.9 (normal), 25.0–29.9 (overweight), and ≥30 (obese) kg/m^2^. The total number of chronic physical conditions (angina, arthritis, asthma, stroke, diabetes, edentulism, cataract, chronic obstructive lung disease, hypertension, hearing problems) was calculated. The participant was considered to have the condition in the presence of self-reported diagnosis and/or symptom-based diagnosis using algorithms (blood pressure in case of hypertension), observation by interviewer (hearing problems), or self-reported conditions (edentulism: loss of all natural teeth). Detailed information on the variables on chronic conditions are provided in S1 and S2 Tables. Details on mobility and pain/discomfort are provided in the section on health status below.

### Mental health

Variables pertaining to this domain included affect, sleep/energy, and cognition. Details on these variables are provided on the section on health status below.

### General health

‘In general, how would you rate your health today?’ was the question used to assess self-rated health. Those who answered ‘bad’ or ‘very bad’ were considered to have poor self-rated health [26]. The 12-item validated version of the World Health Organization Disability Assessment Schedule 2.0 (WHODAS 2.0) was used to assess disability [27], and a scale ranging from 0 (no disability) to 10 (maximum disability) was created [28].

### Health status (mobility, pain/discomfort, affect, sleep/energy, cognition)

Ten health-related questions pertaining to five health domains were used to assess health status: (a) mobility; (b) pain/discomfort; (c) affect; (d) sleep/energy; (e) cognition. Details of these variables are provided in previous publications [29, 30]. Briefly, a scale ranging from 0 to 10 was created with factor analysis with polychoric correlations for each domain based on two questions assessing past 30-day health function (actual questions are provided in S3 Table). Higher scores represented greater levels of impairment in health. The overall correlation coefficients between the two questions in each domain were: mobility (0.57); pain/discomfort (0.89); affect (0.82); sleep/energy (0.79); cognition (0.76).

### Statistical analysis

The statistical analysis was done with Stata 14.1 (Stata Corp LP, College station, Texas). The selection of the 22 correlates of SB was based on past literature [13–15]. We assessed the association between the correlates (exposure) and SB (outcome) with multivariable logistic regression. First, we assessed the sociodemographic correlates of SB by constructing a model which includes all the sociodemographic variables (age, sex, education, wealth, marital status, living arrangement, setting, employment status). Next, we assessed the association between each of the other correlates with SB while adjusting for all the sociodemographic variables mentioned above. Analyses using the overall sample including all countries and country-wise analyses were done. Country adjustment was done in the analysis using the overall sample by including dummy variables for each country. All variables were included in the models as categorical variables with the exception of number of chronic conditions, mobility, pain/discomfort, affect, sleep/energy, cognition, and disability (continuous variables). The sample weighting and the complex study design were taken into account in all analyses. Results from the regression analyses are presented as odds ratios (ORs) with 95% confidence intervals (CIs). The level of statistical significance was set at P<0.05.

## Results

A total of 42,469 (China n = 14,811; Ghana n = 5108; India n = 11230; Mexico n = 2742; Russia n = 4355; South Africa n = 4223) individuals aged ≥18 years were included. The overall mean (SD) age was 43.8 (14.4) and 50.1% were females. The overall prevalence (95%CI) of high SB (i.e., ≥8 hours/day) was 8.3% (7.1–9.7%) with the corresponding country-wise figures being: China 9.0% (7.5–10.9%), Ghana 6.4% (5.1–8.1%), India 5.2% (4.2–6.4%), Mexico 3.9% (2.3–6.5%), Russia 17.7% (11.6–25.9%), and South Africa 4.6% (2.2–9.4%). The overall median (IQR) time spent sedentary per day was 180 (120–300) min. The sample characteristics (overall and by country) are provided in Table 1. Russia had the highest proportion of older people, females, ≥secondary education, urban residents, and people living alone. South Africa and Mexico had a high proportion of obese individuals and people engaged in low levels of physical activity.

**Table 1:** Sample characteristics (overall and by country).

Characteristic	Category	Overall	China	Ghana	India	Mexico	Russia	S. Africa
**Sedentary behavior**	≥8 hours/day	8.3	9.0	6.4	5.2	3.9	17.7	4.6
**Sociodemographics**								
Age (years)	18–44	55.3	49.9	55.4	62.5	63.1	48.5	60.2
	45–64	34.4	40.4	33.4	28.8	26.9	33.6	31.8
	≥65	10.3	9.8	11.2	8.6	9.9	17.9	8.0
Sex	Female	50.1	49.1	50.0	49.1	52.0	55.0	52.8
Education	≥Secondary	56.9	62.5	36.6	38.7	48.4	96.8	62.5
Marital status	Married/cohabiting	80.8	89.0	72.6	81.9	69.7	61.1	52.8
Living arrangement	Alone	5.7	5.8	6.3	0.8	0.7	18.8	9.5
Setting	Urban	44.4	48.5	45.8	25.5	77.7	81.5	69.3
Employment status	Unemployed	38.5	32.3	18.4	44.1	48.3	36.6	59.1
**Health behavior**								
Smoking	Never	60.5	64.3	84.0	55.2	59.4	58.6	69.7
	Current smoker	35.2	32.2	8.2	42.5	25.0	28.9	24.2
	Former smoker	4.3	3.5	7.8	2.3	15.6	12.5	6.1
Alcohol consumption	Never	68.3	66.5	44.8	84.0	45.3	19.2	76.5
	Non-heavy	26.3	24.9	52.5	15.3	45.6	70.8	15.4
	Heavy	5.4	8.6	2.7	0.7	9.1	10.0	8.1
Physical inactivity	Yes	17.9	23.1	15.7	12.5	28.8	11.0	42.0
**Physical health**								
Body mass index (kg/m^2^)	<18.5	16.8	4.1	9.2	36.0	0.9	1.5	3.2
	18.5–24.9	55.3	63.8	56.9	52.6	21.4	41.5	35.3
	25.0–29.9	20.9	27.3	21.8	9.1	49.1	36.6	28.5
	≥30.0	7.0	4.9	12.1	2.3	28.5	20.4	33.0
No. of chronic conditions	Mean (SD)	1.0 (1.2)	0.8 (1.0)	0.8 (0.9)	1.0 (1.3)	1.0 (1.2)	1.3 (1.6)	0.9 (1.0)
Mobility^a^	Mean (SD)	1.8 (2.4)	1.1 (1.8)	2.2 (2.5)	2.5 (2.6)	1.8 (2.5)	2.1 (2.6)	1.4 (2.5)
Pain/discomfort^a^	Mean (SD)	2.0 (2.4)	1.3 (2.0)	2.6 (2.5)	2.6 (2.7)	2.0 (2.4)	1.8 (2.4)	2.0 (2.6)
**Mental health**								
Affect^a^	Mean (SD)	1.6 (2.4)	0.6 (1.6)	2.0 (2.5)	2.6 (2.6)	2.1 (2.4)	1.4 (2.2)	2.2 (2.6)
Sleep/energy^a^	Mean (SD)	1.7 (2.3)	1.1 (1.9)	1.9 (2.5)	2.1 (2.5)	1.6 (2.2)	2.5 (2.4)	1.8 (2.6)
Cognition^a^	Mean (SD)	1.7 (2.4)	1.1 (2.0)	1.9 (2.5)	2.3 (2.6)	1.4 (2.0)	1.5 (2.2)	1.5 (2.4)
**General health**								
Disabilitiy^b^	Mean (SD)	1.0 (1.5)	0.4 (0.8)	1.2 (1.6)	1.6 (1.7)	0.9 (1.4)	1.1 (1.4)	1.1 (1.7)
Self-rated health	Poor	11.3	11.4	9.2	11.3	7.2	11.4	10.0

Abbreviation: SD Standard deviation; S. Africa South Africa

Data are % unless otherwise stated.

Estimates are based on weighted sample.

^a^ Scores range from 0–10 with higher scores indicating worse health status.

^b^ Disability was assessed by WHODAS 2.0 with scores ranging from 0–10. Higher scores indicate higher levels of disability.

In the overall sample, urban setting and unemployment were the only significant correlates of high SB (Table 2). In the individual countries, older age was a significant correlate of high SB only in Ghana, India, and Mexico, while males were more sedentary only in India and South Africa. Higher education was significantly associated with high SB only in Mexico, while the richer were more likely to have high SB in China and Ghana.

**Table 2:** Sociodemographic correlates of highly sedentary behavior^a^ estimated by multivariable logistic regression (overall and by country).

Characteristic	Category	Overall	China	Ghana	India	Mexico	Russia	S. Africa
		OR [95%CI]	OR [95%CI]	OR [95%CI]	OR [95%CI]	OR [95%CI]	OR [95%CI]	OR [95%CI]
Age (years)	18–44	1.00	1.00	1.00	1.00	1.00	1.00	1.00
	45–64	0.81	0.58	0.93	1.16	1.43	0.91	0.99
		[0.60,1.08]	[0.38,0.88]	[0.52,1.66]	[0.78,1.73]	[0.58,3.48]	[0.42,1.98]	[0.37,2.67]
	≥65	1.45	0.88	1.96	3.02	2.79	0.98	1.72
		[0.95,2.20]	[0.60,1.29]	[1.18,3.24]	[2.05,4.46]	[1.15,6.80]	[0.26,3.79]	[0.63,4.72]
Sex	Male vs. Female	1.21	1.18	0.67	1.49	0.87	0.87	5.94
		[0.94,1.56]	[0.87,1.61]	[0.38,1.19]	[1.02,2.17]	[0.24,3.07]	[0.53,1.42]	[1.33,26.52]
Education	≥Secondary vs. Else	1.01	0.90	1.00	1.02	2.37	0.80	0.98
		[0.76,1.36]	[0.56,1.46]	[0.55,1.82]	[0.64,1.63]	[1.36,4.13]	[0.45,1.44]	[0.30,3.23]
Wealth	Poorest	1.00	1.00	1.00	1.00	1.00	1.00	1.00
	Poorer	1.09	1.43	1.68	1.05	0.49	1.08	1.54
		[0.75,1.56]	[0.95,2.16]	[0.87,3.23]	[0.58,1.92]	[0.12,1.90]	[0.52,2.25]	[0.29,8.03]
	Middle	1.29	2.39	1.30	0.65	0.74	1.52	3.60
		[0.79,2.09]	[1.39,4.09]	[0.62,2.72]	[0.34,1.24]	[0.15,3.55]	[0.47,4.94]	[0.65,19.84]
	Richer	0.82	2.02	1.70	0.54	1.27	0.44	0.52
		[0.54,1.24]	[1.18,3.44]	[0.84,3.44]	[0.26,1.11]	[0.30,5.29]	[0.21,0.93]	[0.08,3.23]
	Richest	1.27	3.09	2.81	0.72	2.48	0.70	0.14
		[0.81,2.00]	[1.79,5.34]	[1.16,6.80]	[0.34,1.53]	[0.69,8.92]	[0.23,2.19]	[0.02,1.12]
Marital status	Not Married/cohabiting vs. Else	1.23	1.43	1.19	1.29	2.44	0.83	1.56
		[0.96,1.60]	[0.93,2.22]	[0.69,2.05]	[0.96,1.75]	[0.76,7.85]	[0.56,1.23]	[0.46,5.27]
Living alone	Yes vs. No	1.53	1.61	1.03	0.78	0.92	2.08	0.80
		[0.90,2.60]	[0.97,2.67]	[0.51,2.06]	[0.24,2.48]	[0.30,2.84]	[0.68,6.39]	[0.18,3.67]
Setting	Urban vs. Rural	1.69	1.95	1.07	1.64	5.92	1.07	2.76
		[1.27,2.24]	[1.24,3.07]	[0.61,1.88]	[0.98,2.73]	[2.34,14.97]	[0.54,2.14]	[1.05,7.24]
Unemployment	Yes vs. No	1.53	1.61	1.36	1.39	0.91	1.75	6.10
		[1.11,2.12]	[1.01,2.56]	[0.82,2.25]	[1.01,1.92]	[0.29,2.88]	[0.77,3.95]	[1.50,24.75]

Abbreviation: S. Africa South Africa

Models are adjusted for all variables in the Table. The overall model is additionally adjusted for country.

^a^ Those reporting 8 or more hours per day spent sedentary were considered to be highly sedentary.

In terms of the other correlates, in the overall sample, current smoking, BMI ≥30 kg/m^2^, greater number of chronic conditions, poor self-reported health, higher disability levels, and worse health status in terms of mobility, pain/discomfort, affect, sleep/energy, and cognition were significantly associated with high SB (Table 3). These factors were significant correlates in at least three of the countries with the exception of smoking, BMI, and number of chronic conditions. BMI<18.5 kg/m^2^ was associated with high SB only in South Africa (P<0.05). Former smoking was a significant correlate of high SB only in India and Mexico.

**Table 3:** Correlates (health behavior, physical health, mental health, general health) of highly sedentary behavior^a^ estimated by multivariable logistic regression (overall and by country).

Characteristic	Category	Overall	China	Ghana	India	Mexico	Russia	S. Africa
		OR [95%CI]	OR [95%CI]	OR [95%CI]	OR [95%CI]	OR [95%CI]	OR [95%CI]	OR [95%CI]
**Health behavior**								
Smoking	Never	1.00	1.00	1.00	1.00	1.00	1.00	1.00
	Current smoker	1.49	1.15	0.54	1.37	2.50	2.81	0.65
		[1.06,2.09]	[0.70,1.89]	[0.28,1.06]	[0.90,2.08]	[0.92,6.77]	[1.44,5.49]	[0.21,2.00]
	Former smoker	1.11	0.82	0.80	3.58	5.21	1.16	0.42
		[0.74,1.67]	[0.51,1.33]	[0.40,1.60]	[1.77,7.25]	[1.71,15.88]	[0.65,2.10]	[0.10,1.69]
Alcohol consumption	Never	1.00	1.00	1.00	1.00	1.00	1.00	1.00
	Non-heavy	1.04	0.66	1.19	1.70	1.24	1.61	1.15
		[0.74,1.47]	[0.38,1.13]	[0.69,2.05]	[1.04,2.80]	[0.49,3.13]	[0.80,3.22]	[0.33,4.05]
	Heavy	0.92	0.75	0.23	0.97	0.47	1.41	1.05
		[0.51,1.66]	[0.33,1.69]	[0.08,0.62]	[0.36,2.62]	[0.06,3.87]	[0.53,3.73]	[0.20,5.51]
**Physical health**								
BMI (kg/m^2^)	<18.5	1.11	1.47	0.82	0.83	1.00	1.01	10.37
		[0.79,1.56]	[0.54,4.03]	[0.46,1.45]	[0.62,1.11]	[1.00,1.00]	[0.41,2.50]	[1.36,78.94]
	18.5–24.9	1.00	1.00	1.00	1.00	1.00	1.00	1.00
	25.0–29.9	1.02	1.15	1.58	1.45	0.84	0.60	1.96
		[0.79,1.32]	[0.81,1.63]	[0.80,3.09]	[0.82,2.56]	[0.19,3.70]	[0.41,0.88]	[0.60,6.37]
	≥30.0	1.54	1.37	1.67	1.18	1.85	1.14	2.22
		[1.05,2.28]	[0.74,2.55]	[0.83,3.33]	[0.44,3.21]	[0.41,8.29]	[0.61,2.16]	[0.83,5.95]
No. of chronic conditions	per increase in one condition	1.14	1.15	1.04	1.17	1.10	1.06	1.10
		[1.03,1.26]	[0.95,1.39]	[0.86,1.25]	[1.07,1.28]	[0.81,1.49]	[0.86,1.30]	[0.72,1.67]
Mobility^b^	per unit increase	1.18	1.21	1.10	1.15	1.25	1.16	1.39
		[1.12,1.25]	[1.10,1.32]	[0.97,1.23]	[1.08,1.24]	[1.04,1.51]	[1.04,1.29]	[1.16,1.68]
Pain/discomfort^b^	per unit increase	1.11	1.11	1.04	1.08	1.00	1.12	1.59
		[1.06,1.16]	[1.02,1.21]	[0.94,1.15]	[1.03,1.14]	[0.82,1.22]	[1.03,1.21]	[1.29,1.95]
**Mental health**								
Affect^b^	per unit increase	1.16	1.14	0.94	1.13	1.07	1.26	1.24
		[1.09,1.24]	[1.04,1.24]	[0.85,1.04]	[1.05,1.20]	[0.91,1.26]	[1.07,1.49]	[1.00,1.54]
Sleep/energy^b^	per unit increase	1.15	1.09	1.03	1.10	1.02	1.27	1.28
		[1.10,1.20]	[1.02,1.18]	[0.93,1.15]	[1.04,1.15]	[0.84,1.23]	[1.14,1.41]	[1.04,1.57]
Cognition^b^	per unit increase	1.07	1.02	0.96	1.08	1.16	1.11	1.43
		[1.03,1.13]	[0.94,1.10]	[0.87,1.07]	[1.02,1.15]	[0.93,1.44]	[1.01,1.24]	[1.12,1.82]
**General health**								
Self-rated health	Poor vs. not poor	2.27	2.08	1.41	2.91	1.90	2.06	1.29
		[1.74,2.96]	[1.31,3.29]	[0.90,2.19]	[2.09,4.06]	[0.66,5.45]	[1.10,3.88]	[0.72,2.34]
Disability^c^	per unit increase	1.35	1.33	1.15	1.20	1.36	1.55	1.90
		[1.26,1.44]	[1.17,1.52]	[0.99,1.33]	[1.10,1.31]	[1.11,1.68]	[1.35,1.78]	[1.49,2.41]

Abbreviation: S. Africa South Africa; BMI Body mass index

Models are adjusted for age, sex, education, wealth, marital status, living arrangement, setting, and unemployment. The overall model is additionally adjusted for country.

^a^ Those reporting 8 or more hours per day spent sedentary were considered to be highly sedentary.

^b^ Scores range from 0–10 with higher scores indicating worse health status.

^c^ Disability was assessed by WHODAS 2.0 with scores ranging from 0–10. Higher scores indicate higher levels of disability.

## Discussion

The current study provides a comprehensive overview of data on SB correlates in six LMICs, which collectively account for 43% of the world’s adult population [20, 21]. The overall prevalence of high SB was 8.3%. The overall median amount of SB was 180 min/day. We found that in the overall sample, being unemployed and living in an urban setting were the most important sociodemographic correlates of high SB. In the health-related domains, smoking, physical inactivity, BMI ≥30 kg/m^2^, greater number of chronic conditions, poor self-reported health, higher disability levels, and worse health status in terms of mobility, pain/discomfort, affect, sleep/energy, and cognition were significantly associated with high SB. We also encountered several between-country differences in terms of the correlates.

### Sociodemographic SB correlates

Among the sociodemographic factors, being unemployed was an important sociodemographic correlate of high SB levels overall. One hypothesis that might partially explain this association is that those who are unemployed may also have several chronic physical and mental health conditions, which in our study were associated with higher levels of SB. It might be speculated as well that being employed increases levels of social connectedness, which may lead to less SB and more opportunities for leisure time physical activity [31]. Another consideration is that typical jobs in LMICs often involves manual labor, thus higher physical activity and less SB.

Apart from being unemployed, our study suggests that people living in urban environments were more likely to engage in high SB than those living in rural areas. It might be hypothesized that urban employment (e.g., service-based jobs) is more sedentary than rural employment (e.g., farming). Cities in LMICs are also often more likely to have higher traffic and crime which reduces activity and may promote more sedentary lifestyle. These factors are linked to more motorized transport use and higher risk for depression [32], which in turn, are also linked to SB. Evidence from prospective studies suggests that in cities, the built environment, which can be defined as the totality of places built or designed, including buildings, grounds around buildings, layout of communities, transportation infrastructure and parks and trails [33], is an important determinant of TV viewing time and other screen behaviors [34]. A 4-year follow-up study in Australia [35] identified that adults in low-walkable neighborhoods increased their TV viewing time compared with those who lived in high-walkable neighborhoods. Given that much of the evidence on the relationship of environmental attributes with SB comes from studies in Australia, Belgium, and the USA [34], the full range of these environmental exposures and their impacts on SB remain to be examined in LMICs [36]. Also, differences between countries in LMICs need to be explored in more detail in terms of its association with urbanicity as the association was particularly strong in South Africa and Mexico.

Older age was associated with higher levels of SB in Ghana, India, and Mexico which is consistent with previous data from the general population in Western countries [13]. Given the unfavorable biomarker profile associated with SB in older adults [37], our data add to the need to develop and test interventions to reduce SB in older age in LMICs.

Other socio-demographic factors showed mixed results depending on the country. For example, in contrast with Western studies [13], Indian and South African men were more likely to be highly sedentary. It might be that in these countries, particularly in more rural areas, women are more responsible for the livelihood of the family, thus, spending less time sedentary. Clearly, future research is required to clarify gender differences in SB in the LMIC context. Nonetheless, the present findings suggest that interrupting sedentary time may be especially important for men in India and South Africa.

Our data was in agreement with research conducted in Western countries [38], demonstrating that richer people were more likely to be highly sedentary in China and Ghana. In urban centers of LMICs, a more Western lifestyle may be evident such as the use of more motorized transport, less labor-demanding jobs, and physically undemanding, mostly screen-based leisure, which may account for the higher sedentary levels in richer individuals in these settings.

More research to clarify the between-country differences (e.g., age difference) is needed but it can be hypothesized that other country-specific factors, which were not accounted for in the current analyses, are important for explaining SB. For example, job characteristics, access to sports facilities, socio-cultural beliefs about being physically active, and the level of awareness about the benefits of avoiding SB following public health campaigns should be explored in more detail.

### Health behavior correlates

In agreement with data from Western studies [13, 14], smoking and physical inactivity but not alcohol consumption was associated with being more sedentary. SB, physical inactivity and smoking may be reflecting a clustering of unhealthy lifestyles and behavior which is increasing in LMICs [39]. The observation that high sedentary levels are also associated with physical inactivity may especially be a relevant target for interventions, as recent findings suggest that the hazardous effects of SB may be reduced if people are highly active [22].

### Health-related correlates

The current study suggests that SB is associated with a higher number of chronic conditions, which is in line with the wider high-income literature [13–15]. Given the inability to ascertain temporality with the SAGE cross-sectional design, it is plausible to suggest that multi-morbid people engage in more SB because of the associated mobility restrictions, pain/discomfort, or mental health burden (affect, sleep/energy problems) associated with chronic conditions. It is however equally plausible as well to suggest that prolonged SB may precipitate the development of chronic conditions, pain/discomfort, or mental health problems. Our data do suggest that interventions focusing on reducing SB should consider chronic conditions, pain/discomfort, and mental health problems. In LMICs, chronic pain conditions are leading causes of years lived with disability and a recent meta-analysis demonstrated that 35% of working adults had chronic pain [40]. In addition, in Western populations, it has been established that chronic pain is associated with higher SB levels, possibly due to psychological concerns about falling and lower balance confidence [41]. Previous research has also demonstrated that people with mental health problems may be more likely to have chronic pain [42, 43], which impacts upon mobility [44], and this may predispose the individual to sedentary behavior [41]. Previous research in Western populations has also demonstrated that SB may increase the risk of developing anxiety [6] and depression [5], possibly through increasing inflammatory markers [45]. Another hypothesis is that being sedentary and not engaging in activities may lead to social solitude and withdrawal from interpersonal relationships, both of which have been linked to increased feelings of social anxiety and depression [46]. Cognitive problems were another important mental health correlate of high SB. Cognitive problems are associated with impairments in executive functioning and this can result in an increased risk of falls, mainly in older people. Falls are associated with a fear of falling again and avoidance of activities [47].

There were also some country-specific differences. For example, underweight was associated with high SB only in South Africa. Low body weight may be an indicator of malnourishment or other serious health problems such as HIV, which is highly prevalent in South Africa, and is associated with being more sedentary [48]. Differences in associations between SB and mental and physical health parameters may also be explained by variations in the use of health care services. It is possible for example that differences in out-of-pocket payments may influence our findings to some extent. When out-of-pocket payments are high, people often delay or defer accessing or using services even if they believe to be in need. Out-of-pocket payments account for a large share of total health expenditure in LMICs [49]. In 2010, out-of-pocket expenditure as a percentage of total health expenditure was 35% in China, 28% in Ghana, 62% in India, 47% in Mexico, 36% in the Russian Federation, and 7% in South Africa [49]. Deferring treatment or receiving suboptimal treatment may mean that the underlying conditions leading to SB may be more severe in some countries.

### Limitations and future research

Whilst our data has a number of strengths, it should be considered in the light of some limitations. First, the study is cross-sectional, therefore directionality cannot be deduced. Thus, future prospective research is required to explore the directionality and mediators of the relationships observed in our study. Longitudinal studies will also allow identification of true determinants of SB and clearly separate them from correlates. Second, SB was measured with a self-report questionnaire, which is known to be prone to bias and less accurate than objective assessments [50, 51]. It is well known that self-reported measures can underestimate sedentary levels [52]. For instance, previous self-reported data on SB among older adults in the general population found that high-income adults engaged in 5.3 hours of SB per day, while this figure increased to 9.4 hours per day when relying on objective measures [3]. In addition, since the survey was conducted between 2007 and 2010, it is possible that our results may not reflect the current situation in the countries included in the study. Finally, Given the large number of comparisons made, there is the possibility for Type I errors.

### Conclusions

Our data provides some guidance that in order to reduce the burden of SB, health policy makers in LMICs should focus on unemployed people and those living in urban centers. Our data add additional, albeit cross-sectional evidence to previous concerns that urbanization, which may offer many opportunities in LMICs, including potentially better access to mental and physical health care, can also introduce new hazards such as a sedentary lifestyle and consequently a higher risk for NCD. Finally, national health policies focusing on reducing SB should consider a wide range of physical and mental health barriers.

## Supporting information

S1 Table Questions used to assess self-reported diagnosis. (DOCX)

S2 Table Questions and answer options used for symptoms-based diagnosis. (DOCX)

S3 Table Questions used to assess health status. (DOCX)
