# Supplementary material for: Correlates of sedentary behavior in the general population: A cross-sectional study using nationally representative data from six low- and middle-income countries
Source: PLoS One. 2018 Aug 10;13(8):e0202222. doi: 10.1371/journal.pone.0202222 (PMC6086470; doi:10.1371/journal.pone.0202222)
Supplement: S1 Table — (DOCX) [file pone.0202222.s001.docx]

| **S1 Table** Questions used to assess self-reported diagnosis (not available for edentulism and hearing problems) | |
| --- | --- |
| Condition | Question |
| Angina | Have you ever been diagnosed with angina or angina pectoris (a heart disease)? |
| Arthritis | Have you ever been diagnosed with/told you have arthritis (a disease of the joints, or by other names rheumatism or osteoarthritis)? |
| Asthma | Have you ever been diagnosed with asthma (an allergic respiratory disease)? |
| Cataract | In the last 5 years, were you diagnosed with a cataract in one or both of your eyes (a cloudiness in the lens of the eye)? |
| Chronic obstructive lung disease | Have you ever been diagnosed with chronic lung disease (emphysema, bronchitis, COPD)? |
| Diabetes | Have you ever been diagnosed with diabetes (high blood sugar)? (not including diabetes associated with a pregnancy) |
| Hypertension | Have you ever been diagnosed with high blood pressure (hypertension)? |
| Stroke | Have you ever been told by a health professional that you have had a stroke? |
