# Supplementary material for: Correlates of sedentary behavior in the general population: A cross-sectional study using nationally representative data from six low- and middle-income countries
Source: PLoS One. 2018 Aug 10;13(8):e0202222. doi: 10.1371/journal.pone.0202222 (PMC6086470; doi:10.1371/journal.pone.0202222)
Supplement: S2 Table — (DOCX) [file pone.0202222.s002.docx]

| **S2 Table** Questions and answer options used for symptoms-based diagnosis (not available for diabetes, stroke, and hearing problems, and hypertension was based on blood pressure measurement) | |
| --- | --- |
| Condition | Symptom-based algorithm |
| Angina | Rose questionnaire ^(^Rose GA. The diagnosis of ischaemic heart pain and intermittent claudication in field surveys. Bull World Health Organ. 1962;27:645-58). |
| Arthritis^1^ | Affirmative answers to all four of the following:  1. During the last 12 months, have you experienced pain, aching, stiffness or swelling in or around the joints (e.g., in arms, hands, legs or feet) which were not related to an injury and lasted for more than a month?  2. During the last 12 months, have you experienced stiffness in the joint in the morning after getting up from bed, or after a long rest of the joint without movement?  3. Did this stiffness last for less than 30 minutes?  4. Did this stiffness go away after exercise or movement in the joint? |
| Asthma^1^ | 1. During the last 12 months, have you experienced attacks of wheezing or whistling breathing? (Yes)  **AND**  2. “Yes” to at least one of the following (past 12 months):  (a) Have you experienced an attack of wheezing that came on after you stopped exercising or some other physical activity?  (b) Have you had a feeling of tightness in your chest?  (c) Have you woken up with a feeling of tightness in your chest in the morning or any other time?  (d) Have you had an attack of shortness of breath that came on without an obvious cause when you were not exercising or doing some physical activity? |
| Cataract^2^ | 1. In the last 12 months, have you experienced cloudy or blurry vision? (Yes)  **AND**  2. In the last 12 months, have you experienced vision problems with light, such as glare from bright lights, or halos around lights? (Yes) |
| Chronic obstructive lung disease^1^ | 1. During the last 12 months, have you experienced any shortness of breath at rest (while awake)?  (Yes)  **OR**  2. “Yes” to both of the following (past 12 months):  (a) Have you experienced any coughing or wheezing for 10 minutes or more at a time?  (b) Have you experienced any coughing up of sputum or phlegm on most days of the month for at least 3 months? |
| Edentulism | Affirmative answer to the question: “Have you lost all of your natural teeth?” |
| Hypertension | Blood pressure was measured three times with a one-minute interval with the use of a wrist blood pressure monitor (Medistar Wrist Blood Pressure Model S) and the mean value of the three measurements was calculated. Hypertension was defined systolic blood pressure ≥140 mmHg or diastolic blood pressure ≥90 mmHg. |

^1^ Validated questions (Details can be found in Arokiasamy P, Uttamacharya, Kowal P, Capistrant BD, Gildner TE, Thiele E, et al. Chronic Noncommunicable Diseases in 6 Low- and Middle-Income Countries: Findings From Wave 1 of the World Health Organization's Study on Global Ageing and Adult Health (SAGE). American journal of epidemiology. 2017;185(6):414-28).

^2^ Same algorithm as Garin N, Koyanagi A, Chatterji S, Tyrovolas S, Olaya B, Leonardi M, et al. Global Multimorbidity Patterns: A Cross-Sectional, Population-Based, Multi-Country Study. The journals of gerontology Series A, Biological sciences and medical sciences. 2016;71(2):205-14.
