# Supplementary material for: Correlates of sedentary behavior in the general population: A cross-sectional study using nationally representative data from six low- and middle-income countries
Source: PLoS One. 2018 Aug 10;13(8):e0202222. doi: 10.1371/journal.pone.0202222 (PMC6086470; doi:10.1371/journal.pone.0202222)
Supplement: S3 Table — (DOCX) [file pone.0202222.s003.docx]

| **S3 Table** Questions used to assess health status | |
| --- | --- |
| **Mobility** | (1) Overall in the last 30 days, how much difficulty did you have with moving around? |
|  | (2) Overall in the last 30 days, how much difficulty did you have in vigorous activities, such as running 3 km (or equivalent) or cycling? |
| **Pain and discomfort** | (1) Overall in the last 30 days, how much of bodily aches or pains did you have?  (2) Overall in the last 30 days, how much bodily discomfort did you have? |
| **Affect** | (1) Overall in the last 30 days, how much of a problem did you have with feeling sad, low or depressed?  (2) Overall in the last 30 days, how much of a problem did you have with worry or anxiety? |
| **Cognition** | (1) Overall in the last 30 days, how much difficulty did you have with concentrating or remembering things? |
|  | (2) Overall in the last 30 days, how much difficulty did you have in learning a new task (for example, learning how to get to a new place, learning a new game, learning a new recipe etc.)? |
| **Sleep and energy** | (1) Overall in the last 30 days, how much of a problem did you have with sleeping, such as falling asleep, waking up frequently during the night or waking up too early in the morning? |
|  | (2) Overall in the last 30 days, how much of a problem did you have due to not feeling rested and refreshed during the day (e.g. feeling tired, not having energy)? |
